# Targeted Assembly of Ultrathin NiO/MoS_2_ Electrodes for Electrocatalytic Hydrogen Evolution in Alkaline Electrolyte

**DOI:** 10.3390/nano10081547

**Published:** 2020-08-07

**Authors:** Kai Xia, Meiyu Cong, Fanfan Xu, Xin Ding, Xiaodong Zhang

**Affiliations:** College of Chemistry and Chemical Engineering, Qingdao University, Qingdao 266071, China; xiakaiqd@163.com (K.X.); 2017020817@qdu.edu.cn (M.C.); 2018020313@qdu.edu.cn (F.X.)

**Keywords:** hydrogen evolution, electrocatalyst, synergistic, binder-free, water splitting

## Abstract

The development of non-noble metal catalysts for hydrogen revolution in alkaline media is highly desirable, but remains a great challenge. Herein, synergetic ultrathin NiO/MoS_2_ catalysts were prepared to improve the sluggish water dissociation step for HER in alkaline conditions. With traditional electrode assembly methods, MoS_2_:NiO-3:1 exhibited the best catalytic performance; an overpotential of 158 mV was required to achieve a current density of 10 mA/cm^2^. Further, a synergetic ultrathin NiO/MoS_2_/nickel foam (NF) electrode was assembled by electrophoretic deposition (EPD) and post-processing reactions. The electrode displayed higher electrocatalytic ability and stability, and an overpotential of only 121 mV was needed to achieve a current density of 10 mA/cm^2^. The improvement was ascribed to the better catalytic environment, rather than a larger active surface area, a higher density of exposed active sites or other factors. DFT calculations indicated that the hybrid NiO/MoS_2_ heterostuctured interface is advantageous for the enhanced water dissociation step and the corresponding lower kinetic energy barrier—from 1.53 to 0.81 eV.

## 1. Introduction

The hydrogen economy is expected to offer solutions to various problems associated with environmental pollution and fossil fuel depletion. Electrochemical water splitting to extract hydrogen is considered to be an effective way of satisfying future environmental and economic requirements for fuel sources [1,2,3]. High-performance and cost-effective catalysts for water splitting are a critical factor for hydrogen-based energy technologies. Generally, noble metal-based electrocatalysts such as Pt/C, Ir/C, and RuO_2_ have high activities for water splitting. However, the high cost and scarcity of these elements are barriers to their applications in industrial hydrogen production. Noble metal-free electrocatalysts are commonly considered as the best choice and might address these issues [4,5,6,7,8,9,10,11,12,13,14]. The dissociation of water is the rate-determining reaction in aquatic hydrogen revolution and features a complex and energy intensive pathway [7,15,16,17,18,19], which is most effective in alkaline media [20,21,22,23,24,25]. For most metal catalysts, their hydrogen evolution reaction (HER) activity in alkaline solution is inferior compared to in acid environments, which markedly affects the development of electroactive materials [26,27,28,29,30,31,32,33]. Thus, the development of non-noble metal catalysts for the HER with a high activity for water splitting in alkaline media is of great importance.

As reported, a HER process involves water dissociation (Volmer step, H_2_O + e^−^ → H_ad_ + OH^−^) and H_2_ formation (Heyrovsky step, H_2_O + H_ad_ + e^−^ → H_2_ + OH^−^, or Tafel step, 2H_ad_ → H_2_) in alkaline electrolytes [34,35]. The key difference between HER in acidic and alkaline media is that in an alkaline electrolyte, the kinetics are often limited by the sluggish water dissociation step. Increasing the rate of water dissociation is currently a central problem. Fortunately, the formation of nano-interfaces between multiple phases has been confirmed to promote the water dissociation step in electrocatalysis [34,35,36,37,38,39]. Recently, Zhao et al. fabricated ultrathin transition metal dichalcogenide (MoS_2_, WS_2_)/3D metal hydroxide hybridized nanosheets to enhance HER in alkaline electrolytes [35]. Jiang et al. reported mutually beneficial Co_3_O_4_@MoS_2_ heterostructures, which balanced HER and OER performance by improving the kinetics of both reactions [38]. Sabbaraman et al. reported an enhanced HER activity in alkaline solution by modifying Li^+^–Ni(OH)_2_–Pt interfaces [39]. They also found that the edges of Ni(OH)_2_ facilitated a water dissociation process, which was further promoted through Li^+^-induced destabilization of the HO–H bond. All these works provided basic theories and criterions in synthesizing HER catalysts in alkaline electrolytes. Additionally, apart from effective catalysts, a reasonable pathway to assemble electrodes with low ohmic loss and a large active surface area was also very important and urgent. In a traditional system, catalysts are assembled on the conductive substrate with Nafion [6,40,41,42,43]. This binding agent is acidic and weakly conductive, which affects the catalytic activities of the electrocatalysts by reducing conductivity and blocking active sites. However, few studies have focused on the assembly of synergetic electrodes for HER in alkaline media.

## 2. Materials and Methods

**Scheme 1:**
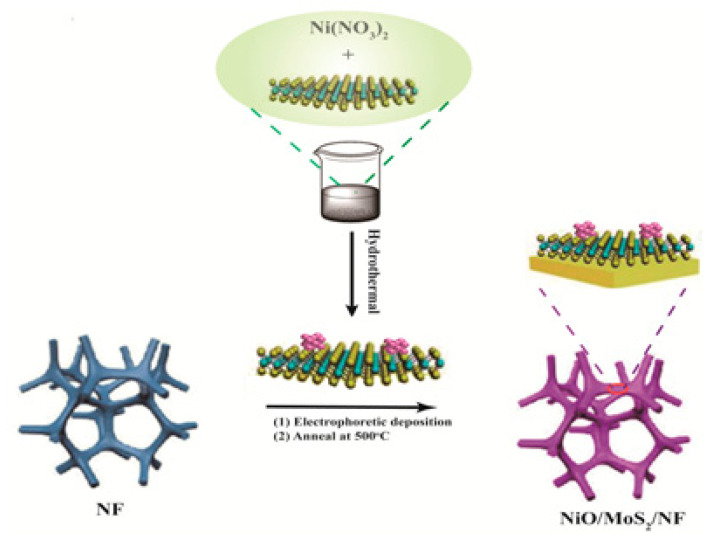
Schematic of the synthesis of a synergetic NiO/MoS_2_ electrode.

Herein, we introduce a simple method of assembling NiO/MoS_2_ electrocatalysts with a facile method. As shown in Scheme 1 and S1, the NiO/MoS_2_ composite catalysts were synthesized with different Ni contents with delaminated MoS_2_ (Figure S1) [molar ratios of MoS_2_:Ni(NO_3_)_2_·6H_2_O], denoted as MoS_2_, MoS_2_:NiO-1:1, MoS_2_:NiO-2:1, MoS_2_:NiO-3:1, and MoS_2_:NiO-4:1, corresponding to the amount of added Ni(NO_3_)_2_·6H_2_O. Among these composite catalysts, MoS_2_:NiO-3:1 exhibited the best catalytic HER performance—an overpotential of 158 mV was required to achieve a current density of 10 mA/cm^2^ in a traditional Nafion-NiO/MoS_2_/GC electrode construction. Furthermore, a NiO/MOS_2_/NF electrode was assembled by electrophoretic deposition (EPD) and post-processing reactions. (Figure S2) This electrode showed a higher electrocatalytic ability and stability than that of the Nafion-based construction and a low overpotential of 121 mV was required to achieve a current density of 10 mA/cm^2^. This higher performance was ascribed to the optimized catalytic environment and faster HER kinetics, rather than a greater active surface area, a higher density of exposed active sites, or other factors. Hence, we established an effective pathway for assembling multi-component electrodes for HER under alkaline conditions. The results and simple synthesis method offer a promising approach to assembling multi-component electrodes for energy storage and conversion devices.

## 3. Results

**Figure 1:**
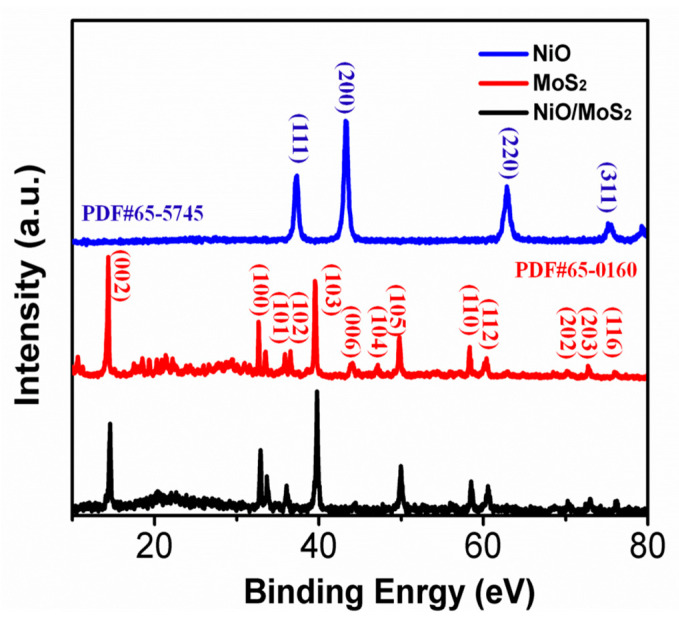
XRD patterns for MoS_2_:NiO-3:1, NiO and MoS_2_.

**Figure 2:**
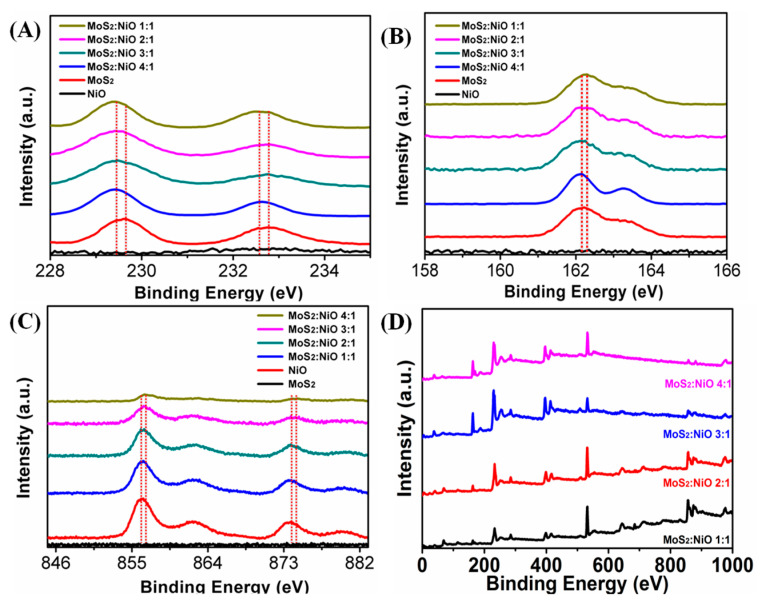
XPS spectra of MoS_2_:NiO catalysts (**D**) in the Mo 3d (**A**), S 2p (**B**) and Ni 2p (**C**) spectral regions.

The X-ray diffraction (XRD) was used to analyze the crystalline structure of the catalyst, as shown in Figure 1. Diffraction peaks at 37.2°, 43.2°, 62.8°, and 75.4° were indexed to the (111), (200), (220), and (311) planes of NiO, respectively (JCPDS No.65-5745). The diffraction peaks at 14.4°, 32.7°, 39.5°, 49.8°, 58.3°, 60.4°, 70.4°, and 72.8° are assigned to the (002), (100), (103), (105), (110), (112), (202), and (203) planes of MoS_2_, respectively (JCPDS No.65-0160). Thus, we identified all characteristic peaks of MoS_2_ in the XRD patterns of MoS_2_:NiO-3:1. Signals from NiO were barely discernable from the baseline, owing to its low content in the composite catalyst. The NiO loading was only 1%, as measured by EDS (Figure S3). We used X-ray photoelectron spectroscopy (XPS) to investigate the components and chemical states of the NiO/MoS_2_ hybrid (Figure 2). For the ultrathin MoS_2_, the Mo 3*d* spectrum consisted of peaks at 229.4 and 232.6 eV, corresponding to the Mo^4+^ 3*d*_5/2_ and Mo^4+^ 3*d*_3/2_ components of MoS_2_ (Figure 2A and Figure S4). In the S 2*p* spectrum, we identified doublet peaks of the 2H phase of MoS_2_, S 2*p*_1/2_, and S 2*p*_3/2_ at 163.4 and 162.2 eV, respectively (Figure 2B and Figure S5). We noted a 0.2 eV negative shift in the binding energy of Mo^4+^ in the results of MoS_2_ and MoS_2_:NiO-1:1. Furthermore, there was a small positive shift in the binding energy of the S 2*p* spectrum after formation of the NiO/MoS_2_ catalyst. Ni species were in a Ni (II) state with Ni 2*p*_1/2_, and Ni 2*p*_3/2_ signals at 856.3 and 873.7 eV, and satellite peaks at 862.5 and 880.6 eV in the XPS spectra of Ni 2*p* (Figure 2C and Figure S6). At higher NiO contents, i.e., MoS_2_:NiO-4:1, there was a small positive shift in the binding energy. The maximum measured difference of 0.2 eV suggested limited charge transfer between NiO and MoS_2_ [35].

**Figure 3:**
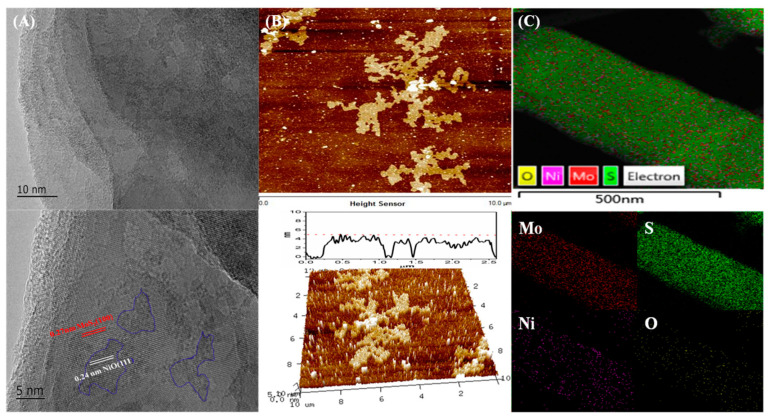
(**A**) HRTEM image of MoS_2_:NiO-3:1; (**B**) AFM of MoS_2_:NiO-3:1; (**C**) elemental mapping of MoS_2_:NiO-3:1, showing a clear uniform distribution of Mo (red), S (green), Ni (purple), and O (yellow). Scale bars are all 500 nm.

The morphologies and structures of the ultrathin MoS_2_:NiO-3:1 were imaged with a transmission electron microscope (TEM) and atomic force microscope (AFM). The high-resolution (HR) TEM images of MoS_2_:NiO-3:1 indicated the formation of NiO nanoparticles, as outlined in blue, which were tightly anchored to the MoS_2_ nanosheets and uniformly dispersed. HRTEM images of the MoS_2_: NiO-3:1 nanosheets showed well-resolved lattice fringes, with an interplane spacing of 0.24 nm corresponding to the (111) plane of NiO and 0.27 nm corresponding to the (100) plane of MoS_2_. The HRTEM images (Figure 3A and Figure S7) and AFM (Figure 3B and Figure S8) suggested that the thickness of the monolithic laminated construction was less than 5 nm and consisted of five MoS_2_ layers. Elemental mapping images also indicated a homogeneous distribution of Ni and O, as shown in Figure 3C. From XRD, XPS, HRTEM and AFM, we confirmed that NiO was prepared on ultrathin MoS_2_, forming a NiO/MoS_2_ catalyst. From our TEM and AFM imaging, we further confirmed the formation of a synergetic NiO/MoS_2_ catalyst.

**Figure 4:**
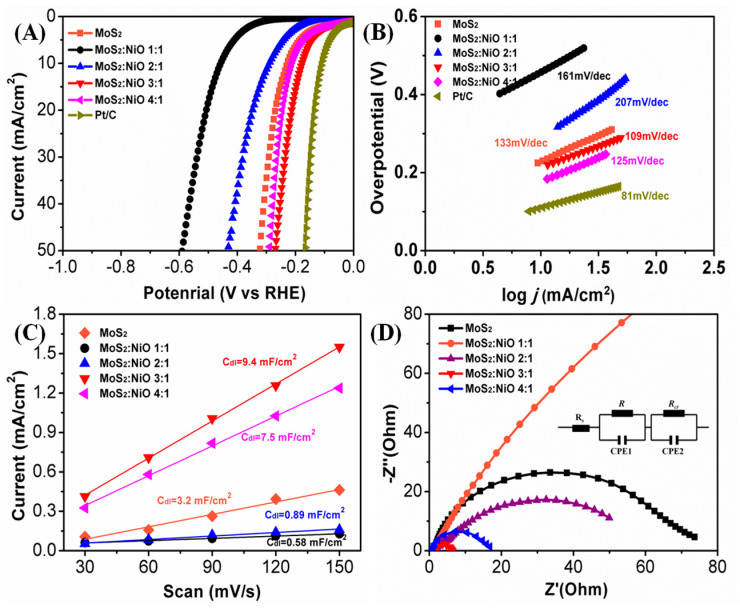
HER electrocatalytic activity of NiO/MoS_2_ catalysts: (**A**) polarization curves after *iR* compensation; (**B**) Tafel plots of electrodes at a scan rate of 5 mV/s; (**C**) capacitive current at 0.165 V as a function of the scan rate; (**D**) EIS curves of electrodes at an overpotential of 200 mV.

The electrocatalytic performance was performed in a standard three-electrode system at a scan rate of 5 mV/s in a 1.0 M KOH alkaline aqueous solution. First, the electrodes were assembled in a traditional system, and the catalyst was assembled on a glass carbon electrode (GC) with Nafion. The Nafion-NiO/MoS_2_/GC electrodes acted as a working electrode, and carbon rods and a Ag/AgCl electrode as the counter electrode and reference electrode, respectively. Owing to the influence of ohmic resistance, the as-measured reaction currents do not directly reflect the inherent performance of the catalysts, therefore we applied iR compensation to all raw data, and all potentials were recorded on a reversible hydrogen electrode (RHE) scale in this work. From the linear sweep voltammetry (LSV) curves (Figure 4A), we found that the Pt/C has excellent HER activity and only required an overpotential of 109 mV to achieve a current density of 10 mA cm^−2^. This performance ranks second only to that of electrode of Pt/C. The MoS_2_:NiO-3:1 electrocatalyst had the lowest overpotential (η) of approximately 158 mV among that of MoS_2_ (228 mV), MoS_2_:NiO-1:1 (457 mV), MoS_2_:NiO-2:1 (290 mV), and MoS_2_:NiO-4:1 (213 mV). As plotted in Figure 4B, the Tafel slope of MoS_2_:NiO-3:1 was 109 mV/dec, which was lower than that of other catalysts [MoS_2_ (133 mV/dec ), MoS_2_:NiO-1:1 (161 mV/dec), MoS_2_:NiO-2:1 (207 mV/dec), and MoS_2_: NiO-4:1 (125 mV/dec)]. This result suggests that the HER activity can be tuned by changing the NiO content. The decrease in the Tafel slope indicates that the HER kinetics were improved by growth of NiO on ultrathin MoS_2_. Figure 4C and Figures S9–S13 show the corresponding capacitive current at 0.165 V as a function of the scan rate, which gives a double-layer capacitance (C_dl_) of 9.4 mF/cm^2^ for MoS_2_:NiO-3:1, which is greater than that of the MoS_2_ (3.2 mF/cm^2^), MoS_2_:NiO-1:1 (0.58 mF/cm^2^), MoS_2_:NiO-2:1 (0.89 mF/cm^2^), and MoS_2_:NiO-4:1 (7.5 mF/cm^2^) electrode systems. Thus, a greater active surface area is one reason for the higher catalytic activity of the MoS_2_:NiO-3:1 electrode system. We used electrochemical impedance spectroscopy (EIS) to investigate the HER kinetics of these catalysts. The Nyquist plots were fitted with the equivalent circuit inset in Figure 4D and Figure S14. The plots were fitted by a model with two parallel constant phase elements: one high frequency component related to surface porosity (R) and a low frequency component related to R_ct_ [44,45]. The value of R_ct_ was assigned as the charge transfer resistance at the interface between the catalyst and electrolyte, which is generally used to represent the electrochemical activity of an electrode. At an overpotential of 300 mV, the R_ct_ value of MoS_2_:NiO-3:1 was much lower than that of all other catalysts. Hence, ultrathin MoS_2_ modified with a moderate amount of NiO greatly reduced the charge-transfer resistance, which accelerated the HER kinetics.

**Figure 5:**
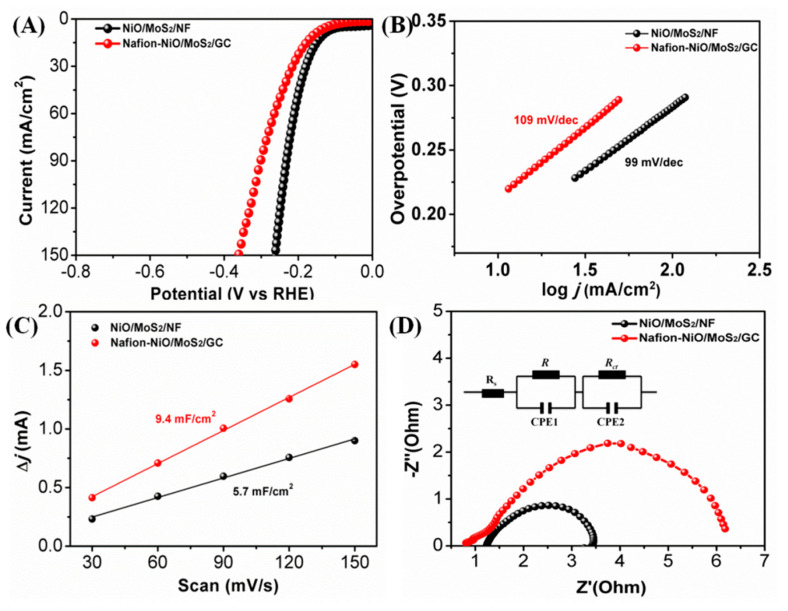
Electrochemical performance for HER. (**A**) J–V curves of representative assembling conditions: NiO/MoS_2_/NF and Nafion-NiO/MoS_2_/GC; (**B**) Tafel plots for Nafion–NiO/MoS_2_/GC and NiO/MoS_2_/NF; (**C**) capacitive current at 0.165 V as a function of the scan rate; (**D**) EIS curves of electrodes at an overpotential of 200 mV.

We focused our attention on the most active catalyst system, MoS_2_:NiO-3:1, and compared the results of the Nafion-NiO/MoS_2_/GC and binder-free NiO/MoS_2_/NF systems from the LSV curves in the electrocatalytic hydrogen evolution reaction at a scan rate of 5 mV/s. The catalyst loading of the binder-free NiO/MoS_2_/NF was controlled through the assembly time over a EPD period at a steady current density of 5 mA/cm^2^. The quantity of Mo was accurately measured by inductively coupled plasma atomic emission spectrometry (ICP-AES), as shown in Table S1. After 5 min of EPD, the NiO/MoS_2_/NF had the best performance (Figures S15 and S16, Table S1), with a low catalyst loading of 0.072 mg/cm^2^, which was lower than that of the Nafion-NiO/MoS_2_/GC system (0.106 mg/cm^2^). As shown in Figure 5A, an overpotential of 121 mV was required to achieve a current density of 10 mA/cm^2^ for the binder-free NiO/MoS_2_/NF, which was lower than that of NiO/MoS_2_/GC (158 mV). Thus, our synthesis method yielded high-performance electrodes for HER. To confirm the enhanced HER activity of NiO/MoS_2_/NF, a low Tafel slope of 99 mV/dec was measured together with a smaller C_dl_ value of 5.7 mF/cm^2^ at a low catalyst loading (Figure S17), superior than most reported electrodes (Table S2). Thus, improved HER kinetics were the main factor contributing to the performance of this binder-free NiO/MoS_2_/NF, which had a smaller active surface area and a lower density of exposed active sites compared with those features of the Nafion-based catalyst. Additionally, as shown in Figure 5D, Nyquist plots showed a considerably lower charge transfer resistance for NiO/MoS_2_/NF, owing to opening of pathways with greatly reduced charge-transfer resistance in the absence of the weakly conductive Nafion membrane. Hence, the higher electrocatalytic activities of NiO/MoS_2_/NF were confirmed to arise from accelerated HER kinetics and reduced charge-transfer resistance in the optimized catalytic environment rather than a larger active surface area, a higher density of exposed active sites or other factors.

**Figure 6:**
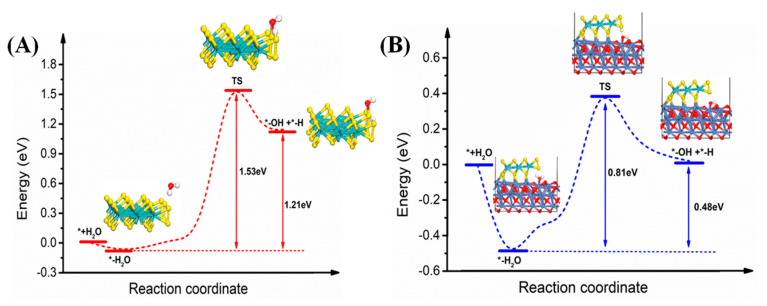
Energy pathway for water dissociation at the MoS_2_ zigzag edge (**A**) and the MoS_2_/NiO (111) interface (**B**) with atomic configurations of initial state (water adsorption), transition state (TS) and final state (water dissociation). The reaction energy and the activation barrier are indicated in the inset.

We also performed density functional theory (DFT) calculations to reveal the contributions of the hybrid NiO/MoS_2_ heterostructured interface to the enhanced HER performance. Because the basal plane of MoS_2_ is catalytically inert to HER, we focused on the Mo edge. Optimized structures of MoS_2_ with a zigzag edge (i.e., a Mo edge saturated with S) and the hybrid MoS_2_/NiO (111) are shown in Figure S18, and the corresponding reaction pathways for H_2_O dissociation over MoS_2_ and the MoS_2_/NiO (111) hybrid are presented in Figure 6. At the MoS_2_/NiO (111) interface, strong chemical bonding between S and the surface under coordinated Ni was observed, which resulted in severe reconstruction of the Ni (111) surface. From Figure 6, we note that H_2_O adsorption at the MoS_2_ edge is very weak (an adsorption energy of −0.06 eV). Moreover, the dissociation of H_2_O into adsorbed H and OH over the MoS_2_ edge is energetically endothermic, requiring an input of approximately 1.21 eV, and has an extremely high energy barrier (1.53 eV) to overcome; hence, water dissociation at pure MoS_2_ is thermodynamically unfavorable. However, with the assistance of NiO, the H_2_O adsorption becomes stronger at surface Ni sites (−0.46 eV), and the reaction energy and the kinetic activation barrier of H_2_O dissociation decrease to 0.48 and 0.81 eV, respectively. In the dissociated state, H is bound to the S edge, whereas OH is bonded to the surface Ni. Thus, both the thermodynamics and kinetics of the water dissociation step (i.e., the rate-determining step of HER under alkaline conditions) can be effectively promoted by hybridization of MoS_2_ with NiO, which is in good agreement with our experimental observations.

## 4. Conclusions

In conclusion, ultrathin NiO/MoS_2_ catalysts were prepared to promote the water dissociation step for HER in alkaline conditions. The combined experimental measurements and DFT calculations suggest that the hybrid NiO/MoS_2_ heterostructured interface was responsible for the enhanced water dissociation step and corresponding lower kinetic energy barrier. With the use of traditional electrode assembly methods, the MoS_2_:NiO-3:1 catalyst exhibited the best catalytic HER performance, and an overpotential of 158 mV was required to achieve a current density of 10 mA/cm^2^. Furthermore, a binder-free NiO/MoS_2_/NF electrode was also assembled by EPD and post-processing reactions. This electrode had better electrocatalytic performance and stability, with an overpotential of only 121 mV required to achieve current density of 10 mA/cm^2^, which is clearly superior to the performance of the traditional electrodes. We attribute these improvements to the better catalytic environment and faster HER kinetics. Thus, we have introduced an effective pathway for assembling synergetic multi-component electrodes for HER under alkaline conditions for the first time. These results and the simple synthesis method offer a promising direction for assembling synergetic multi-component electrodes for energy storage and conversion devices.

## Supplementary Materials

The following are available online at https://www.mdpi.com/2079-4991/10/8/1547/s1, Scheme S1: Schematic of the synthesis of synergetic NiO/MoS_2_ electrode, Figure S1: (A) The SEM image of pure MoS_2_. (B)SEM of the MoS_2_ after exfoliation, Figure S2: (A, B) Different resolution SEM images of NiO/MoS_2_/NF electrode, Figure S3: The EDX mapping for NiO/MoS_2_ catalyst powder, Figure S4: XPS spectra of Mo 3d regions of NiO/MoS_2_ catalysts, Figure S5: XPS spectra of S 2*p* regions of NiO/MoS_2_ catalysts, Figure S6: XPS spectra of Ni 2*p* regions of NiO/MoS_2_ catalysts, Figure S7: HRTEM image of MoS_2_:NiO-3:1, Figure S8: AFM of MoS_2_:NiO-3:1, Figure S9: CVs of MoS_2_:NiO 3:1 (A) and corresponding Cdl (B), Figure S10: CVs of MoS_2_:NiO 4:1 (A) and corresponding Cdl (B), Figure S11: CVs of MoS_2_ (A) and corresponding Cdl (B), Figure S12: CVs of MoS_2_:NiO 2:1 (A) and corresponding Cdl (B), Figure S13: CVs of MoS_2_:NiO 1:1 (A) and corresponding Cdl (B), Figure S14: EIS curves of NiO/MoS_2_/NF at different overpotential from 100~300 mV, Figure S15: LSV curves for NiO/MoS_2_/NF under various electrophoresis time, Figure S16: LSV curves for NiO/MoS_2_/NF before and after 500 CV cycle (left); Stability test of the NiO/MoS_2_/NF electrode at a fixed current density of 10 mA/cm^2^ for HER, Figure S17: CV curves for NiO/MoS_2_/NF, Figure S18: Optimized structures of MoS_2_ with zigzag edge (a) and the hybrid MoS_2_/NiO(111) (b, side view). Color modes: yellow for S, green for Mo, red for O and cyan for Ni, Table S1: The Overpotential of different electrodes at 10 and 50 mA/cm^2^, Table S2: A representative summary of HER performances of nonprecious materials based electrocatalysts previously reported catalyst for HER in 1M KOH aqueous solution.
