# Supplementary material for: Targeted Assembly of Ultrathin NiO/MoS2 Electrodes for Electrocatalytic Hydrogen Evolution in Alkaline Electrolyte
Source: Nanomaterials (Basel). 2020 Aug 7;10(8):1547. doi: 10.3390/nano10081547 (PMC7466591; doi:10.3390/nano10081547)
Supplement: Supplementary file 1 [file nanomaterials-10-01547-s001.pdf]

Supporting Information

Targeted Assembly of Ultrathin NiO/MoS_2 Electrodes for Electrocatalytic Hydrogen Evolution in Alkaline Electrolyte

Kai Xia, Meiyu Cong, Fanfan Xu, Xin Ding,* and Xiaodong Zhang*

College of Chemistry and Chemical Engineering,Qingdao University, Qingdao 266071, Shandong, P. R.China; xiakaiqd@163.com (K.X.); 2017020817@qdu.edu.cn (M.C.); 2018020313@qdu.edu.cn (F.X.);

* Correspondence: dingxin@qdu.edu.cn (X.D.); zhangxd@hotmail.com (X.Z.)

Experimental Procedures

Chemicals

Commercial bulk MoS_2, $Ni(NO_3)_2{\cdot}6H_2O$ and NF (nickel foam) were purchased from Aladdin Ltd. (Shanghai, China). All reagents were analytical reagent grade without further purification. Ultrapure water (Millipore Milli-Q grade) with a resistivity of 18.2 MΩ was used in all experiments.

Synthesis of MoS_2 Nanosheets

20 mg bulk MoS_2 was dispersed in 20 ml dimethyl formamide (DMF) and stripped by ultrasonic cell disruptor for 3 h. Subsequently, the resulting dispersion was centrifuged for 10 min at 10000 rpm and the supernatant was decanted gently, the remaining sediment was cleaned by deionized water until reaching neutral and dried in a vacuum at 70 °C for 6 hours. After cooling naturally, the MoS_2 nanosheets were obtained.

Synthesis of Different Molar Ratio NiO/MoS_2 Hybrid Materials

Firstly, specific mass (8 mg, 16 mg, 24 mg, 32 mg) of MoS_2 nanosheets above-mentioned were dissolved into 50 mL 1 mmol/L $Ni(NO_3)_2$ solution respectively. Next, these solutions were ultrasonic mixed for 10 min separately. Then the above solution was transferred to Teflon-lined stainless-steel autoclaves and heated in an electric oven at 120 °C for 6 h. After cooling naturally, the black precipitates were obtained by ultrasonic cell disruptor, washed with distilled water and ethanol several times, and dried in a vacuum at 70 °C for 3 h. Subsequently, the samples were heated to 500 °C for 2 h under Ar atmosphere with a heating rate of 4 °C/min. After cooling to ambient temperature, the different mixed (the molar ratio of MoS_2 : NiO was 1:1, 2:1, 3:1, 4:1) NiO/MoS_2 materials was obtained.

Preparation of Nafion-NiO/MoS_2/GC Electrode

To fabricate Nafion-NiO/MoS_2/GC electrode, 1.5 mg of each different mixed NiO/MoS_2 materials and 10 μL of Nafion solution (5 wt%) were dispersed in 190 μL deionized water/ethanol solvent (V:V = 1:1) with sonication for 1 h. Then 10 μL catalyst ink was loaded on bare GC electrode (3 mm in diameter) with a catalyst loading of about 0.106 mg/cm^2.

Preparation of NiO/MoS_2/NF Electrode

After the electrochemical measurements of different molar ratio, we found that the catalyst performed best when the molar ratio was 3:1. So we fabricated the NiO/MoS_2/NF electrode with NiO/MoS_2-3:1 to explore effects of the assembly method on HER electrochemistry activity. In detail, 2 mg NiO/MoS_2-3:1 power was dispersed in 10 mL DMF and vibrated with ultrasonic wave for 1 h to fabricate homogeneous solution. Then 1 mL 50 μg/mL DMF solution of $Ni(NO_3)_2$ was added in

above-mentioned dispersion to use for electrophoresis. After electrophoresising with a steady current of 5 mA for 5 min, The NF was heated to 500 °C for 2 h under Ar atmosphere with a heating rate of 4 °C/min. Cooling down to room temperature, then we obtained the NiO/MoS_2/NF electrode.

Characterizations

Crystallographic structure of all as-prepared samples was investigated with X-ray powder diffraction (XRD, X'Pert PRO MPD, CuKR) at a scanning rate of 1 °C/min. XRD data were collected in the 2θ ranges from 10º to 80º. The morphology of the samples were examined with field-emission scanning electron microscopy (SEM, Hitachi, S-4800). Transmission electron microscopy (TEM) images were collected on HRTEM, JEM-2100UHR with an accelerating voltage of 200 kV. The samples were prepared by dropping the ethanol solution of samples on the Cu grids and were observed at 100 kV. The X-ray photoelectron spectroscopy (XPS) measurements were performed in an ESCALAB 250 spectrometer. EDX elemental mapping were performed on JEOL ARM-200F and atomic force microscopy (AFM) were implemented by Bruker Dimension Icon system. We found that the catalyst performed best and its performance was the nearest with platinum after electrochemistry tests when the molar ratio was 3:1, so we tested characterizations of this specific proportion powder.

Electrochemical measurements

The electrochemical performance of electrodes were obtained in a standard three-electrode electrochemical cell by AUTOLAB PGSTAT302N electrochemical workstation (Metrohm, Switzerland). The prepared integrated electrodes, carbon rods and saturated Ag/AgCl electrode were used as working electrode, counter electrode and reference electrode, respectively. All the electrochemical tests were carried out in KOH (1.0 mol/L, pH = 13.6) electrolyte.

All potentials reported in this work were calibrated to reversible hydrogen electrode (RHE) according to the Nernst equation ($E_{vs.RHE}$=0.059×pH+$E^{\theta}_{Hg/HgO}$ + $E_{vs.\ Hg/HgO}$ (0.098) or $E_{vs.RHE}$=0.059×pH+$E^{\theta}_{Ag/AgCl}$ + $E_{vs.Ag/AgCl}$ (0.197)). The potentials reported in this work were corrected for the ohmic losses according to $E_{corrected} = E_{measured} - j \times R_s \times A/1000$, where j (mA/cm^2) is the geometric current density, R_s (Ω) is the equivalent series resistance that can be determined from the EIS, and A (cm^2) is the geometric area of the electrode.

The electrochemical activities of samples towards HER were examined by linear sweep voltammetry (LSV) with a scan rate of 5 mV/s at room temperature. It is worth noting that Hg/HgO was used as reference electrode in the stability test for stability consioderation. Tafel slopes were evaluated based on a steady-state current density method and were calculated by plotting overpotential against Log (*j*, current density), the Tafel equation: Z = blog *j* + a, where b is the Tafel slope and a is a constant. The electrochemical impedance spectroscopy (EIS) measurements of samples were performed out in the same configuration from 10^5 to 0.01 Hz with an AC voltage of 5 mV with electrochemical workstation.

Cyclic Voltammetry (CV) taken at various scan rates (30, 60, 90, 120 and 150 mV/s) were recorded in the non-faradic potential range of 0.10-0.23 V vs RHE and were used to estimate the double-layer capacitance (C_{dl}). C_{dl} was determined as the linear slope by plotting anodic current density at 0.165 V against the scan rate. $C_{dl} = i_c/\nu$, Where i_c represents the charging current, ν is the scan rate.

Computations Methods

The DFT calculations were performed to investigate the adsorption and dissociation of water on MoS_2 and MoS_2/NiO(111) by using the Vienna ab initio simulation package (VASP).[1] The ion-electron interaction is described with the projector augmented wave (PAW) method.[2] Electron exchange-correlation is represented by the functional of Perdew, Burke and Ernzerhof (PBE) of generalized gradient approximation (GGA).[3] A cutoff energy of 450 eV was used for the plane-wave basis set. The MoS2 edge was modeled in a zigzag nanoribbon with width of three Mo-S chains, and

the Mo edge is 100%-saturated by S. The NiO(111) surface slab was modeled in a rectangular $3\sqrt{3}\times3$ unit mesh with six-layer thickness. The bottom three layers were kept fixed at the optimized bulk positions during all the computations, and the thickness of the vacuum layer was set to be 12 Å to ensure the decoupling between periodic images. The Brillouin zone was sampled by a Monkhorst-Pack k-point mesh of 2×4×1 grid. The convergence threshold for structural optimization was set to be 10^{-5} eV in energy and 0.01 eV/Å in force. The climbing-image nudged elastic band (CI-NEB) method [4] was used to determine the minimum energy pathways for H_2O dissociation, and the transition states were obtained by relaxing the force below 0.05 eV/Å.

Results

Characterization Atlas

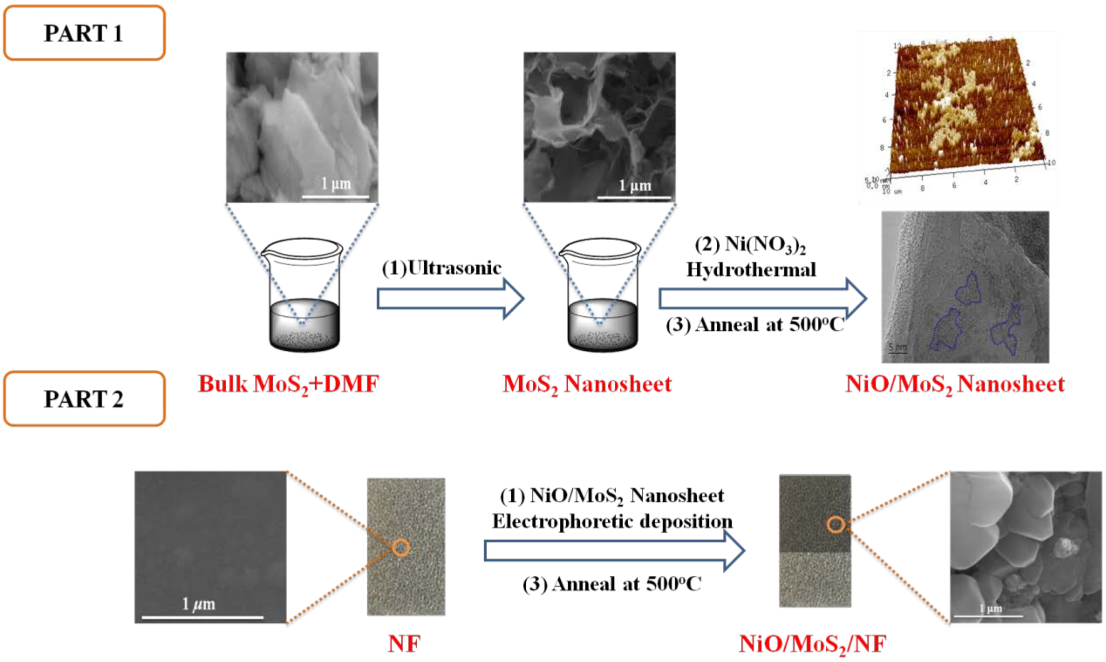

Scheme S1. Schematic of the synthesis of synergetic NiO/MoS_2 electrode.

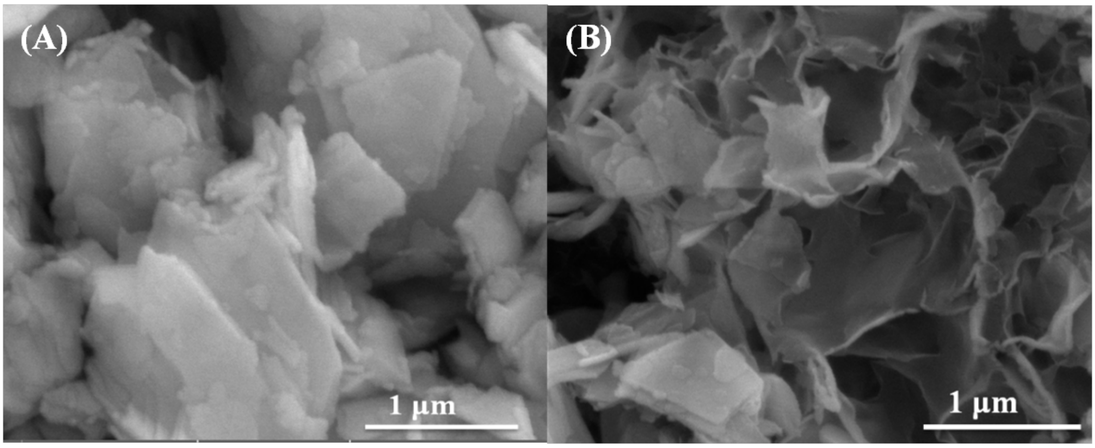

Figure 1. (A) The SEM image of pure MoS_2. (B)SEM of the MoS_2 after exfoliation.

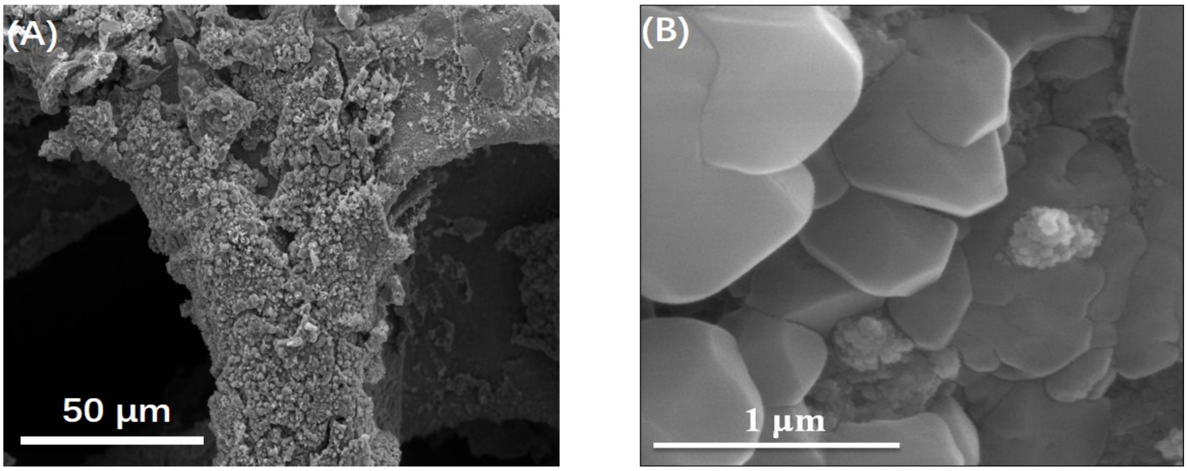

Figure 2. (A, B) Different resolution SEM images of NiO/MoS_2/NF electrode.

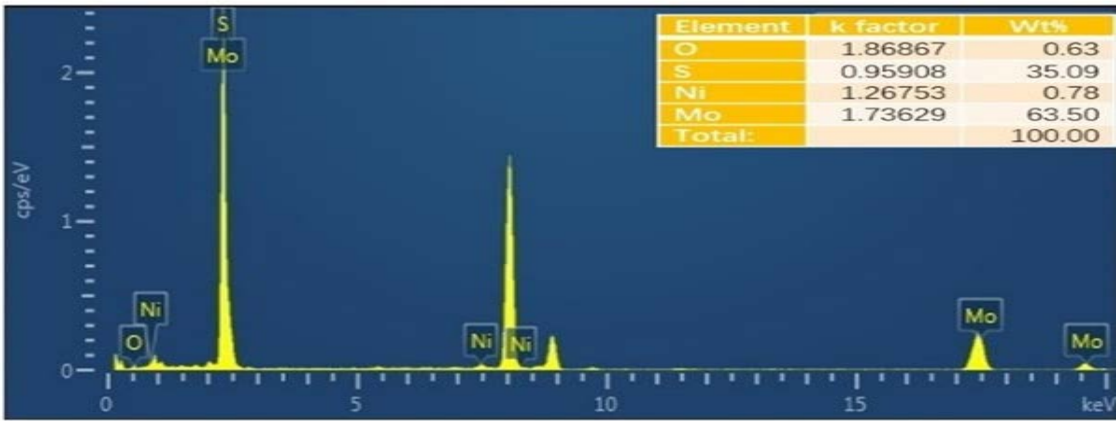

Figure 3. The EDX mapping for NiO/MoS_2 catalyst powder.

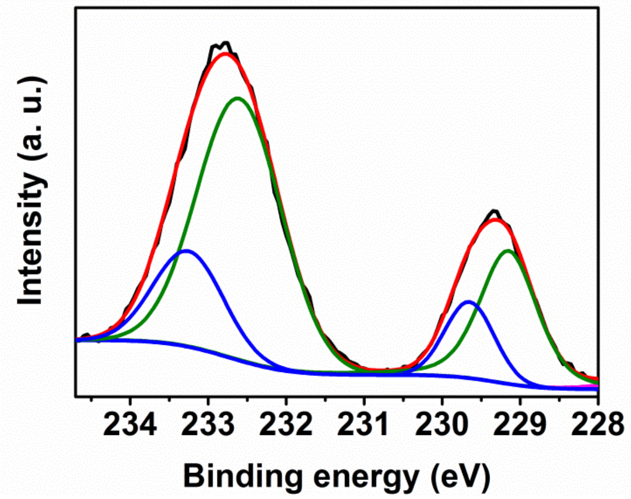

Figure 4. XPS spectra of Mo 3*d* regions of NiO/MoS_2 catalysts.

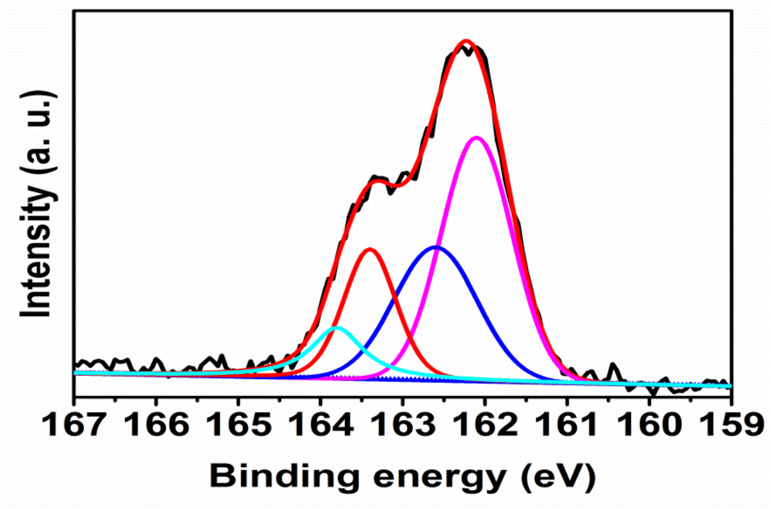

Figure 5. spectra of S 2*p* regions of NiO/MoS_2 catalysts.

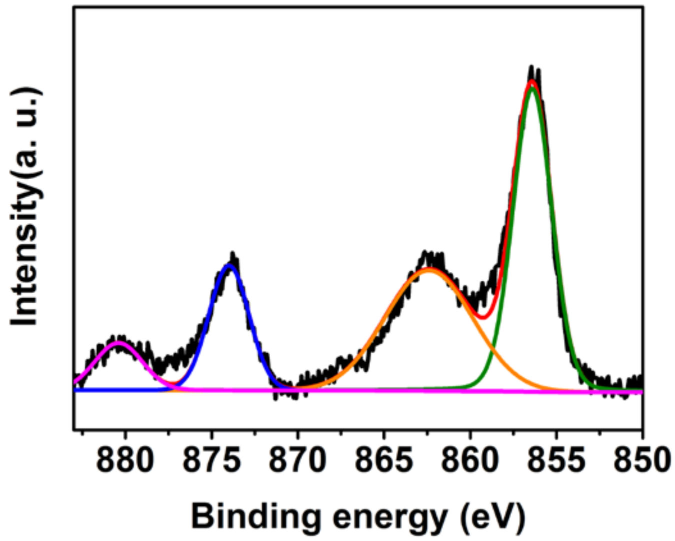

Figure 6. XPS spectra of Ni 2*p* regions of NiO/MoS_2 catalysts.

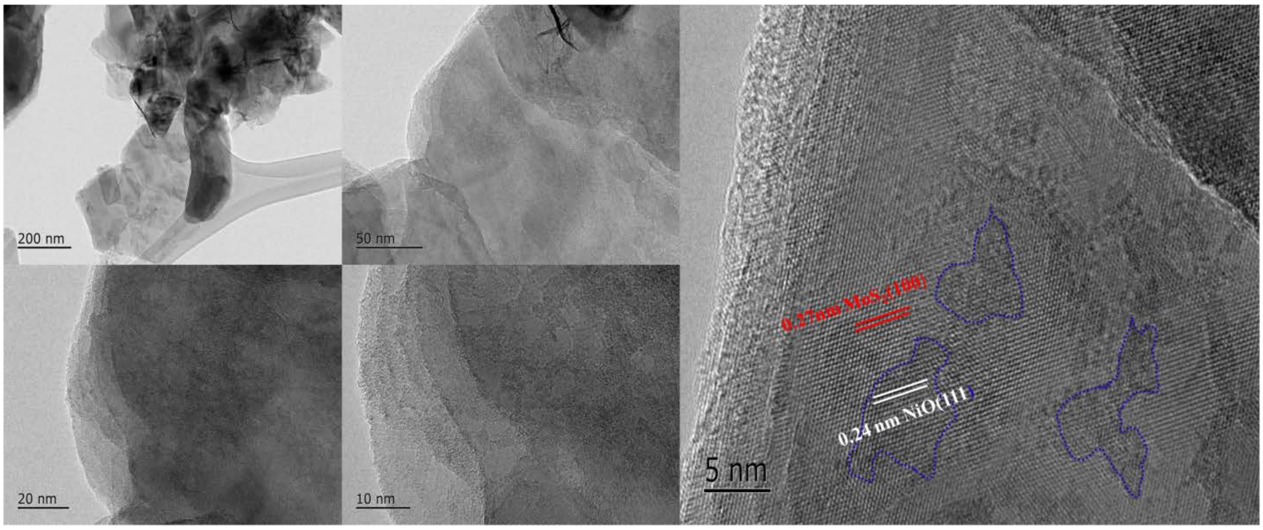

Figure 7. HRTEM image of MoS_2:NiO-3:1.

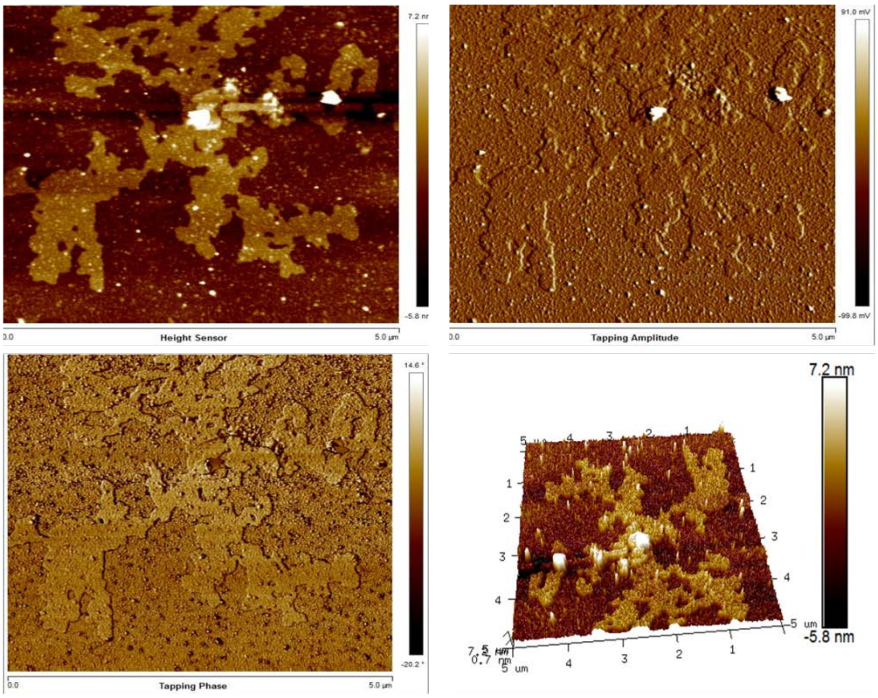

Figure 8. AFM of MoS_2:NiO-3:1.

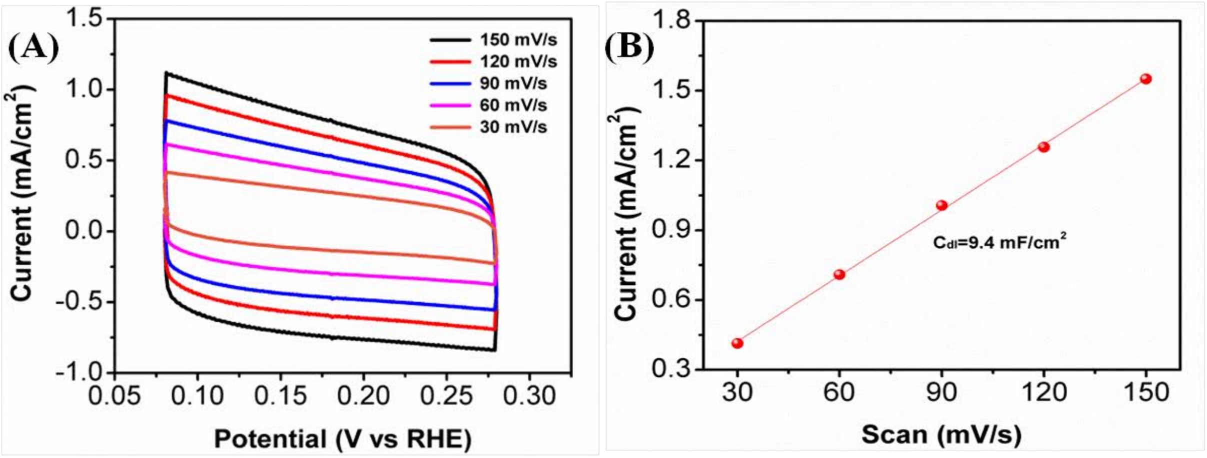

Figure 9. CVs of MoS_2:NiO 3:1 (A) and corresponding C_{dl} (B).

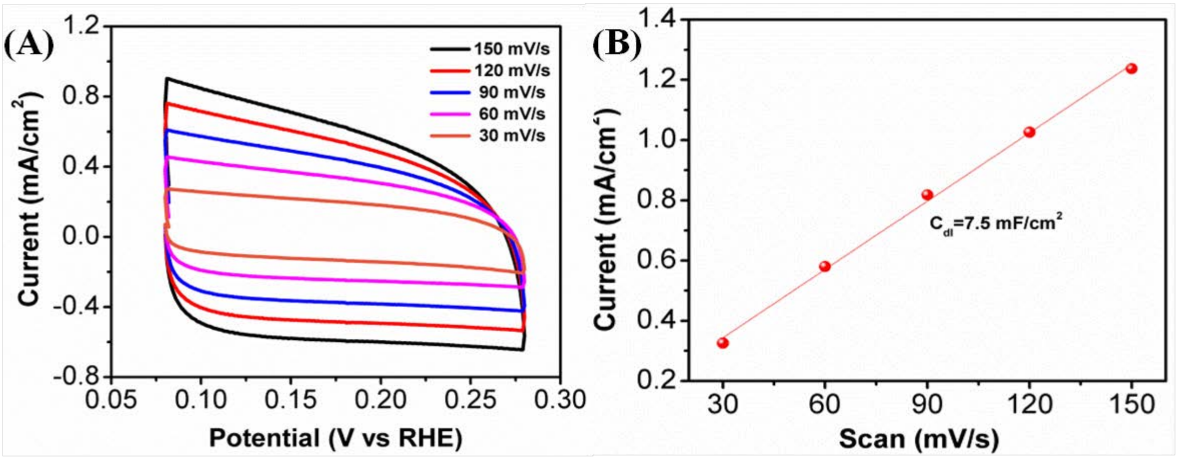

Figure 10. CVs of MoS_2:NiO 4:1 (A) and corresponding C_{dl} (B).

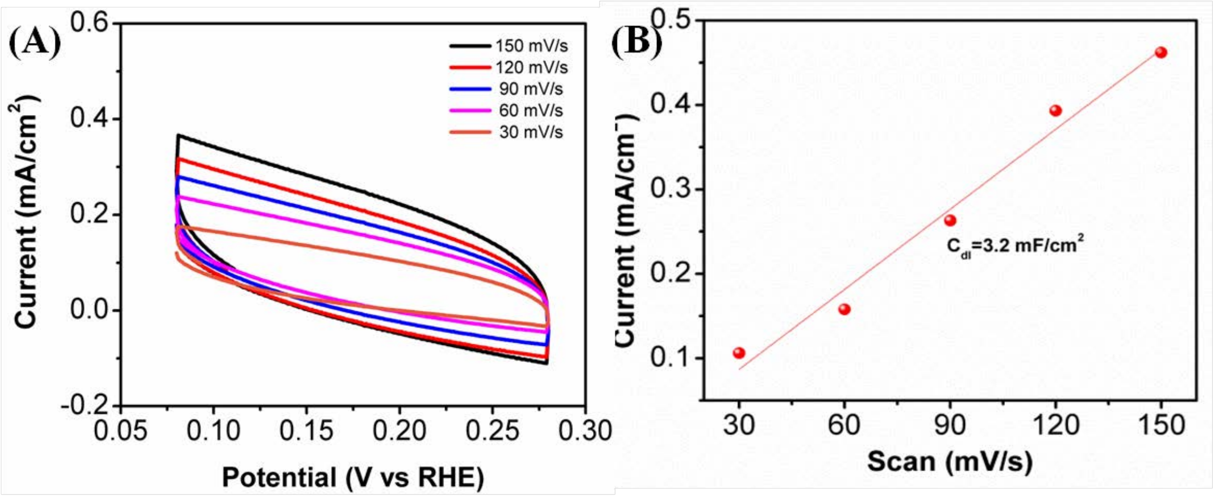

Figure 11. CVs of MoS_2 (A) and corresponding C_{dl} (B).

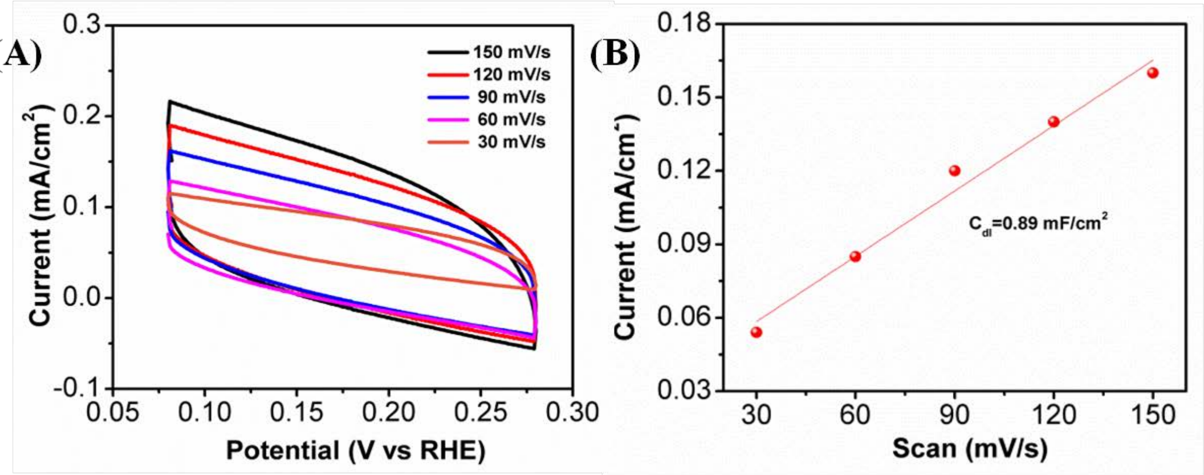

Figure 12. CVs of MoS_2:NiO 2:1 (A) and corresponding C_{dl} (B).

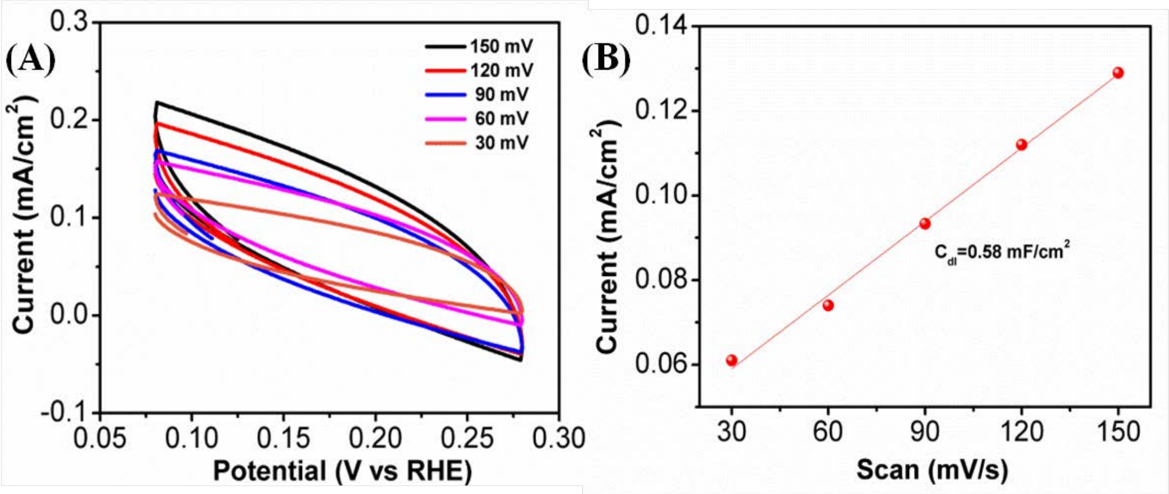

Figure 13. CVs of MoS_2:NiO 1:1 (A) and corresponding C_{dl} (B).

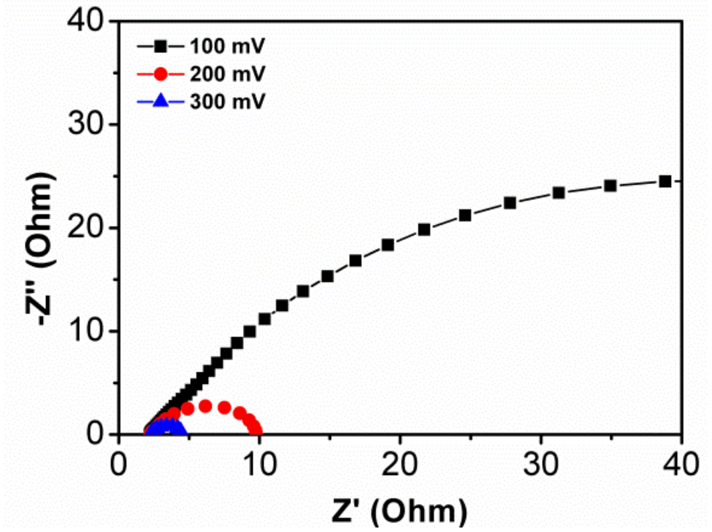

Figure 14. EIS curves of NiO/MoS_2/NF at different overpotential from 100~300 mV.

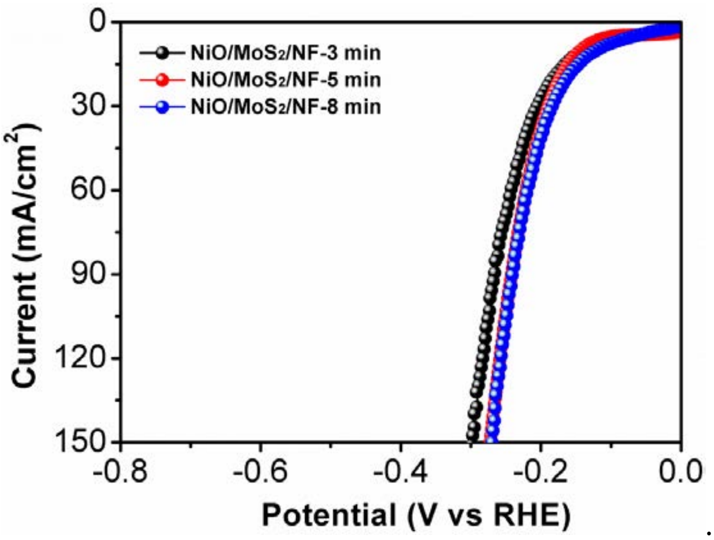

Figure S15. LSV curves for NiO/MoS_2/NF under various electrophoresis time.

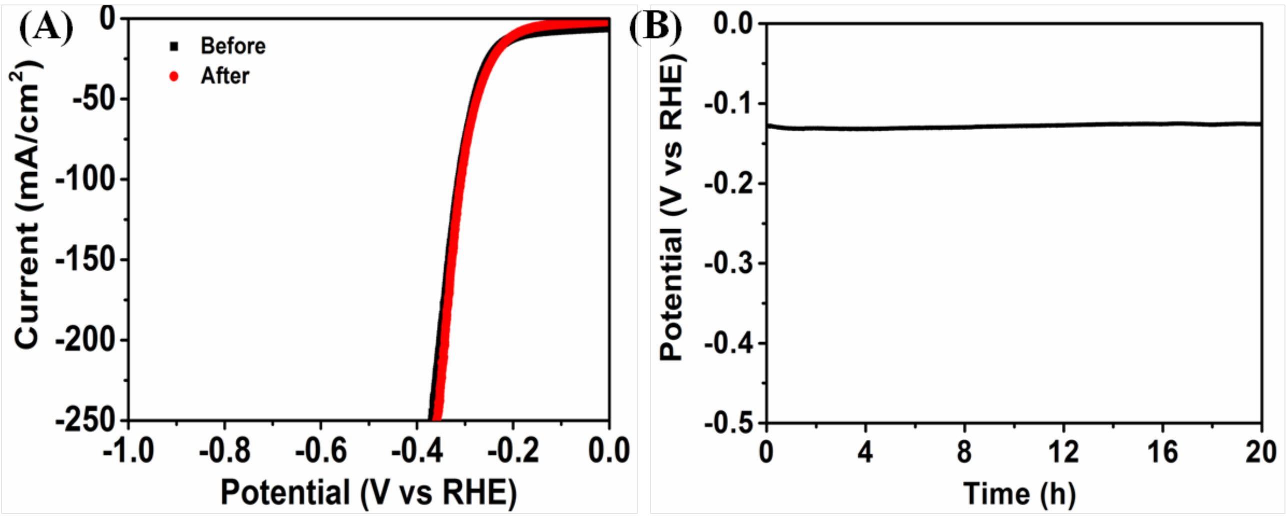

Figure 16. LSV curves for NiO/MoS_2/NF before and after 500 CV cycle (left); Stability test of the NiO/MoS_2/NF electrode at a fixed current density of 10 mA/cm^2 for HER.

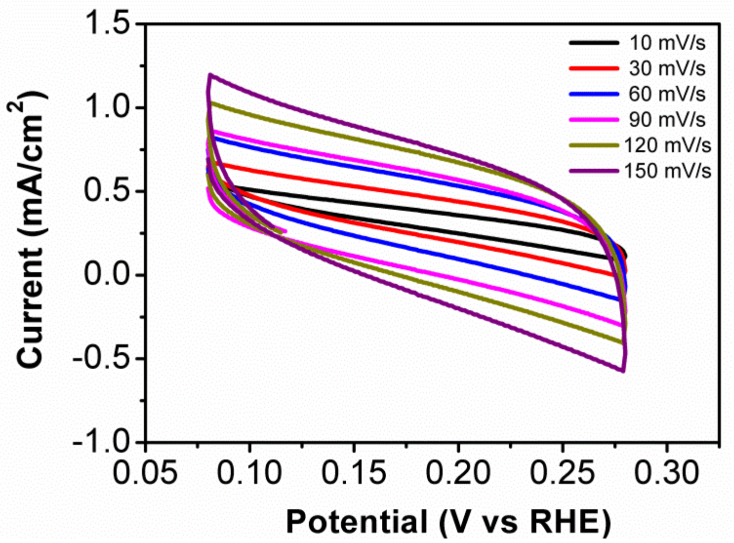

Figure 17. CV curves for NiO/MoS_2/NF.

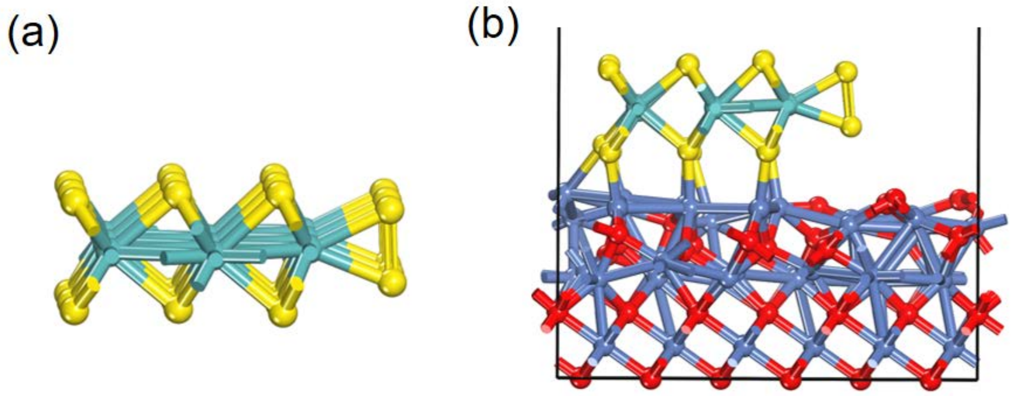

Figure 18. Optimized structures of MoS_2 with zigzag edge (a) and the hybrid MoS_2/NiO(111) (b, side view). Color modes: yellow for S, green for Mo, red for O and cyan for Ni.

Table 1. The Overpotential of different electrodes at 10 and 50 mA/cm^{2}.

Electrode		MoS_2:NiO-3:1 /loading	Overpotential (at 10 mA/cm^2)	Overpotential (at 50 mA/cm^2)
**NiO/MoS_2/NF-5 min**	8 min	0.122 mg/cm^2	143 mV	233 mV
	5 min	0.072 mg/cm^2	121 mV	210 mV
	3 min	0.048 mg/cm^2	145 mV	212 mV
Nafion-NiO/MoS_2/GC	/	0.106 mg/cm^2	158 mV	271 mV

Table 2. A representative summary of HER performances of nonprecious materials based electrocatalysts.

previously reported catalyst for HER in 1M KOH aqueous solution .

Catalyst	Overpotental (at 10 mA/cm^2)	Reference
VOOH	221 mV	Angew Chem Int Ed Engl,2017, 56 573-577.
MoS_2	351 mV	Advanced Material 2018, 1801171

2D-MoS_2/$Ni(OH)_2$-10	185 mV	Advanced Material 2018, 1801171
2D-MoS_2/$Co(OH)_2$-10	125 mV	Advanced Material 2018, 1801171
Ni-Co-MoS_2 Nanoboxes	155 mV	Advanced Material 2016, 28, 9006-9011
Mo_2C/OMC-3	175 mV	ACS Applied Energy Material 2018,1,736-743
1T MoS_2/$Ni^{2+\delta}O_\delta(OH)_{2-\delta}$	185 mV	Advanced Science 2018, 5,1700644
Co_2P@Co_3O_4	159 mV	Journal of Power Sources, 374 (2018) 142-148
CoN/Co	160 mV	Catalysis Science & Technology, 8 (2018) 3695-3703
MoB/g-C_3N_4 Interface Material	133 mV	Angew.Chem. Int.Ed. 2018,57, 496-500
Co_3O_4@MoS_2/CC	207 mV	Journal of Materials Chemistry A,2015,5,2067-2072
1T MoS_2/COOH	160 mV	Nanoscale,2018, 10, 12330–12336
Co/CoP-5	253 mV	Advanced Energy Materials, 2017, 7, 1602355
N-Ni_3S_2/VS_2	151mV	Electrochimica Acta, 2018, 269, 56-61
**NiO/MoS_2/GC**	**158 mV**	**This work**
**NiO/MoS_2/NF**	**121 mV**	**This work**

References

[1] Kresse, G.; Furthmuller, J. *Phys. Rev. B* **1996**, *54*, 11169-11186.

[2] Blochl, P. E. *Phys. Rev. B* **1994**, *50*, 17953-17979.

[3] Perdew, J. P.; Burke, K.; Ernzerhof, M. *Phys. Rev. Lett.* **1996**, *77*, 3865-3868.

[4] Henkelman, G.; Uberuaga, B. P.; Jonsson, H. *J. Chem. Phys.* **2000**, *113*, 9901-9904.
